# Protective Effects of Alginate and Chitosan Oligosaccharides against *Clostridioides difficile* Bacteria and Toxin

**DOI:** 10.3390/toxins15100586

**Published:** 2023-09-22

**Authors:** Maria Eleni Mavrogeni, Mostafa Asadpoor, Jo H. Judernatz, Ingrid van Ark, Marc M. S. M. Wösten, Karin Strijbis, Roland J. Pieters, Gert Folkerts, Saskia Braber

**Affiliations:** 1 Division of Pharmacology, Utrecht Institute for Pharmaceutical Sciences, Faculty of Science, Utrecht University, Universiteitsweg 99, 3584 CG Utrecht, The Netherlands; 2 Structural Biochemistry Group, Bijvoet Centre for Biomolecular Research, Utrecht University, Universiteitsweg 99, 3584 CG Utrecht, The Netherlands; 3 Division of Infectious Diseases and Immunology, Department of Biomolecular Health Sciences, Faculty of Veterinary Medicine, Utrecht University, Yalelaan 1, 3584 CL Utrecht, The Netherlands; 4 Division of Medicinal Chemistry and Chemical Biology, Utrecht Institute for Pharmaceutical Sciences, Faculty of Science, Utrecht University, Universiteitsweg 99, 3584 CG Utrecht, The Netherlands

**Keywords:** *C. difficile*, TcdA, AOS, COS, intestinal epithelial barrier, TJs, TEER, LY, calcium-switch assay

## Abstract

*Clostridioides difficile* infection is expected to become the most common healthcare-associated infection worldwide. *C. difficile*-induced pathogenicity is significantly attributed to its enterotoxin, TcdA, which primarily targets Rho-GTPases involved in regulating cytoskeletal and tight junction (TJ) dynamics, thus leading to cytoskeleton breakdown and ultimately increased intestinal permeability. This study investigated whether two non-digestible oligosaccharides (NDOs), alginate (AOS) and chitosan (COS) oligosaccharides, possess antipathogenic and barrier-protective properties against *C. difficile* bacteria and TcdA toxin, respectively. Both NDOs significantly reduced *C. difficile* growth, while cell cytotoxicity assays demonstrated that neither COS nor AOS significantly attenuated the TcdA-induced cell death 24 h post-exposure. The challenge of Caco-2 monolayers with increasing TcdA concentrations increased paracellular permeability, as measured by TEER and LY flux assays. In this experimental setup, COS completely abolished, and AOS mitigated, the deleterious effects of TcdA on the monolayer’s integrity. These events were not accompanied by alterations in ZO-1 and occludin protein levels; however, immunofluorescence microscopy revealed that both AOS and COS prevented the TcdA-induced occludin mislocalization. Finally, both NDOs accelerated TJ reassembly upon a calcium-switch assay. Overall, this study established the antipathogenic and barrier-protective capacity of AOS and COS against *C. difficile* and its toxin, TcdA, while revealing their ability to promote TJ reassembly in Caco-2 cells.

## 1. Introduction

*Clostridioides difficile* is a Gram-positive, anaerobic, spore-forming, and toxin-producing bacillus known as the most prevalent causative pathogen of nosocomial diarrhea. *C. difficile* infection (CDI) has been estimated to cause 15–25% of all antibiotic-associated diarrhea cases [1,2]. CDI is projected to become the most common healthcare-associated infection worldwide, even surpassing the methicillin-resistant *Staphylococcus aureus* (*S. aureus*) [3,4]. Transmission usually occurs via the fecal–oral route and after reaching the human intestine; the opportunistic bacterium can change into its vegetative disease-causing state. The colonic anaerobic environment and the presence of cholate and glycine derivatives facilitate its colonization and spores’ germination. Under homeostatic conditions, balanced gut microbiota populations inhibit *C. difficile* growth by further processing the cholate derivatives [5]. However, upon induction of dysbiosis, principally due to the consumption of broad-spectrum antibiotics, the disruption of gut flora allows *C. difficile* germination and proliferation [5,6]. Aside from antibiotically induced dysbiosis, CDI development is also associated with other risk factors, including advanced age, pregnancy, acid suppression medications, hypoalbuminemia, and impaired adaptive immunity [2,3,5]. Clinical manifestations of CDI range from asymptomatic colonization, mild diarrhea, abdominal cramps, and fever to fulminant and pseudomembranous colitis, toxic megacolon, bowel perforation, sepsis, and, ultimately, death [2,4].

The pathogenic effects of *C. difficile* are mainly secondary to the activity of two exotoxins, toxin A (TcdA) and toxin B (TcdB), while certain hypervirulent strains produce the binary toxin *C. difficile* transferase (CDT) [5]. Once reaching the cytoplasm of their host target cells, these toxins act as enzymes resulting in the typical clinical manifestations [7]. TcdA and TcdB are the major virulence factors of *C. difficile* and belong to the family of large clostridial glucosylating toxins [7,8]. Both TcdA and TcdB are large, single-unit toxins that act via the mono-glycosylation of small GTP-binding proteins involved in the organization of cytoskeletal dynamics, and their combined action results in colonic tissue inflammation and massive colonic fluid secretion, i.e., watery diarrhea [9,10]. Glycosylation of Rho (Rho, Rac, and Cdc42) proteins halts their interaction with downstream effectors and blocks Rho-dependent signaling pathways, thus causing cytopathic and cytotoxic effects. TcdA possesses glucosyltransferase activity, with a primary target of A-GTPase, a Ras homolog family member (RhoA-GTPases), which is a crucial regulator of actin cytoskeleton dynamics and tight junction assembly. TcdA inactivates RhoA-GTPase by inducing its transition from the guanosine triphosphate (GTP)-bound form (active) to the guanosine diphosphate (GDP)-bound form (inactive), ultimately leading to cytoskeletal breakdown and tight junction (TJ) disassembly [11]. Each TJ is formed by the assembly of various proteins (mainly ZO-1, -2, -3, occludin, claudins, and JAMs) and is located near the apical side of the lateral membrane of the epithelium. Disruption of TJs, the “gate-keepers” of the paracellular route and critical components of the epithelial barrier, results in impaired epithelial barrier integrity, known as the “leaky gut” condition [12]. Increased intestinal permeability leads to acute colonic mucosa inflammation and excessive fluid loss, while the increased invasion of pathogens into the systemic circulation subsequently results in systemic inflammation and initiation of numerous diseases [13].

Even though TcdA and TcdB share a common mechanism of action, i.e., irreversible inactivation of Rho family GTPases that leads to cytoskeletal rearrangement, disruption of cell adhesion junctions, cytopathic effects, and apoptosis, their effect on barrier damage seems to differ [14]. In a study using Caco-2 monolayers, exposure to TcdA resulted in a rapid, progressively decreasing transepithelial electrical resistance (TEER) trend. However, exposure to TcdA and TcdB combined did not have an additional impact on the rate of barrier disruption compared to TcdA alone. Moreover, TcdB required double the time to achieve the same TEER drop compared to an equal TcdA concentration. These findings signified a decreased ability of TcdB to affect TJs and suggested that TcdA plays the main role in the disruption of the epithelial barrier. Finally, the authors showed that TcdA increased the apical to basolateral translocation of TcdB by 100%, while TcdB did not seem to enhance the translocation of TcdA. These observations suggested that TcdA facilitates the translocation of TcdB from the gut into submucosal areas, where it elicits inflammatory responses [15]. Such findings agreed with previous studies suggesting that TcdA and TcdB might act synergistically [1]. As the main interest of this study was the C. *difficile*-induced disruption of the epithelial barrier, we focused on TcdA rather than TcdB, due to its superiority in causing epithelial TJ disruption.

**Figure 1:**
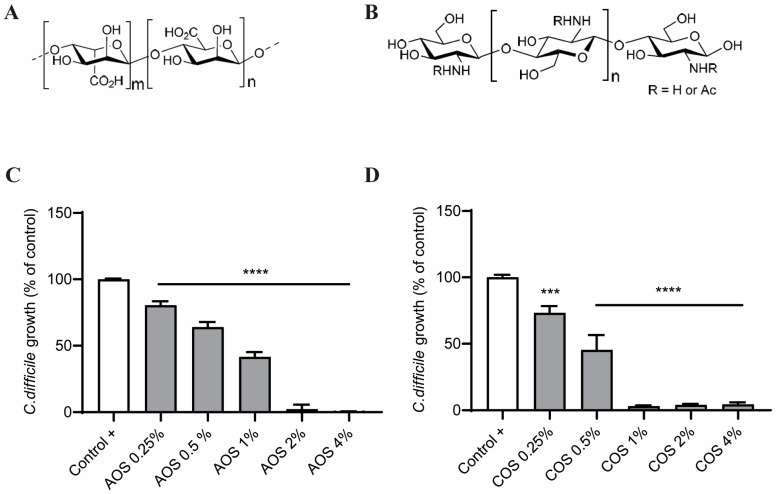
Structures of AOS and COS and MIC assay. (**A**) AOS are composed of β-(1,4)-D-mannuronic acid and α-(1,4)-L-guluronic acid, (**B**) COS are composed of β-(1,4)-GlcNAc and β-(1,4)-GlcN. (**C**,**D**) Effect of AOS and COS on bacterial growth of C. difficile: To examine the anti-growth capacity of the NDOs against C. difficile, an MIC assay was performed for (**C**) AOS and (**D**) COS using six 2-fold serial dilutions of each NDO. Control + represents the percentage of maximal bacterial growth without any treatment. Results are expressed as the percentage of the relative to the control-bacterial growth, as mean ± SEM of two independent experiments, each performed in triplicate. (*** *p* ≤ 0.001 and **** *p* ≤ 0.0001: significantly different from the control +, as obtained using a one-way ANOVA test).

CDI not only causes high mortality but also carries an increased financial burden, especially with the development of antibiotic-resistance mechanisms and disease recurrences [5,16]. At present, available therapeutic strategies for CDI include the use of specific antibiotics against *C. difficile*, fecal transplantation, and surgery. However, severe and recurrent CDI treatment is still exigent, with limited treatment options available [11]. Both *C. difficile* and TcdA constitute appealing pharmacological targets; thus, identifying agents with antibacterial capacity against *C. difficile* or with barrier-protective properties against the TcdA-induced toxicity, or even better with both abilities, are very promising strategies. In this context, the interest in nutraceuticals, particularly in the prebiotic dietary fibers named non-digestible oligosaccharides (NDOs), has recently increased [12]. NDOs possess multiple biological properties and confer innumerable health benefits via their ability to shape intestinal microbiota and the microbiota-related immune responses. Emerging evidence suggests that NDOs also exert their health-beneficial effects microbiota independently by interacting directly with intestinal epithelial and immune cells [17]. Among the numerous NDO categories identified, alginate oligosaccharides (AOS) and chitin/chitosan oligosaccharides (COS) have drawn tremendous attention due to their versatile health-beneficial activities, including antimicrobial and intestinal barrier-reinforcing capabilities [18]. AOS are acidic anionic oligosaccharides obtained upon the degradation or microbial fermentation of alginate, a biopolymer isolated from the cell walls of brown algae [19,20,21]. They are built up of 2–25 monomers with an average molecular weight (MW) of 1.5 kDa and are composed of two types of uronic acid: (1 → 4)-linked-α-L-guluronic acid (G) and (1 → 4)-linked-β-D-mannuronic acid (M) [19,22] (Figure 1A). The monomers form either homo-oligomeric blocks (GG-/MM-blocks) or hetero-oligomeric mixed sequences (GM-blocks) [21,23,24]. COS are prepared from the deacetylation and degradation of chitin or the depolymerization of chitosan [25,26,27]. They consist of β-(1 → 4)-linked N-acetyl-2-amino-2-deoxyglucose (N-acetyl-D-glucosamine/GlcNAc) and β-(1 → 4)-linked D-glucosamine (GlcN) [25,28,29] (Figure 1B). Chitin, which has a high proportion of GlcNAc monomers (degree of acetylation (DA) > 70%), is present in the exoskeletons of crustaceans and arthropods and the cell walls of fungi, algae, and yeast. By contrast, chitosan is mostly composed of GlcN monomers (DA < 30%) and can be extracted from the cell walls of specific fungi [21,25,30]. COS usually have an average MW < 3.9 kDa, a degree of polymerization (DP) of <20, and a high degree of deacetylation (DD) of >90% [21,25,26,29]. The structural characteristics of AOS and COS, e.g., MW, DP, DD, sequence, and N-acetylation pattern, tremendously affect the exhibited physicochemical and, ultimately, biological properties [22,25,26].

Recently, accumulating evidence supports the theory that both AOS and COS exert barrier-protective effects against various triggers known for inducing increased epithelial permeability and leaky gut condition. Among them are inflammatory cytokines (i.e., TNF-α and INF-γ), lipopolysaccharide (LPS), dextran sulfate sodium (DSS), and enterotoxigenic *Escherichia coli* (*E. coli*) [20,24,27,31,32]. Both NDOs exert epithelial barrier protection via their prebiotic activity, i.e., by restoring imbalanced gut flora populations and abundance of their health-beneficial metabolites, short-chain fatty acids (SCFA) [23,33,34,35,36,37]. Apart from their microbiota-related barrier-protective activity, both NDOs have been reported to reinforce the intestinal barrier via direct interactions with intestinal epithelial cells, saving the integrity of TJ complexes responsible for maintaining a functional barrier [20,23,32,38,39,40].

Here, the hypothesis that AOS and COS exert a protective role in TcdA-induced epithelial injury was tested using the Caco-2 cell line as a model for the human intestinal barrier. In addition, AOS [41,42,43], but mostly COS [44,45,46,47,48,49], have been shown to exert various direct effects on exotoxin-producing bacteria, including *Escherichia coli*, *Streptococcus agalactiae*, and *Staphylococcus aureus*, mediated by various mechanisms such as inhibition of bacterial growth, biofilm formation, and adhesion mechanisms [21]. Therefore, in this study, we also examined the growth inhibition of AOS and COS against *C. difficile* by performing a minimum inhibitory concentration (MIC) assay. Both compounds showed great potential in protecting the epithelial layer from TcdA-induced damage and demonstrated growth-inhibiting capacity against *C. difficile*.

## 2. Results

### 2.1. AOS and COS Reduce C. difficile Growth

To examine whether AOS and COS possess anti-growth activity against *C. difficile*, bacterial growth was investigated in the absence or presence of ascending NDO concentrations. As shown in Figure 1C, the addition of AOS induced a significant decrease in the growth of *C. difficile*. This effect was evident already from the concentration of 0.25%. No growth was observed when 2% AOS were added. COS presented a similar pattern: COS 0.25–0.5% significantly suppressed *C. difficile* growth, while at 1% no growth was visible (Figure 1D). This means that the minimum inhibitory concentration for AOS was 2% and for COS it was 1%. Gram-staining and MALDI-TOF analysis confirmed that the cultures contained *C. difficile* (Supplementary Figures S1 and S2).

### 2.2. NDOs and TcdA Concentration Selection

An MTT cell viability assay was conducted to select three concentrations per NDO for the subsequent experiments. After 48 h of exposure to 0–4% AOS or COS, the highest concentration of both NDOs (4%) significantly decreased cell viability in Caco-2 cells (Supplementary Figure S3A,B), thus the concentrations chosen for both AOS and COS were 0.5, 1, and 2%. Furthermore, the cells were triggered with 1 pg/mL–1 μg/mL TcdA and incubated for 24 h. The first three higher concentrations (10 ng/mL–1 μg/mL) significantly reduced cell viability (Supplementary Figure S3C). Three concentrations were selected for further experiments: 100 pg/mL and 1 ng/mL (non-cytotoxic), and 10 ng/mL (cytotoxic).

### 2.3. Exposure of the Caco-2 Monolayer to TcdA Fails to Elicit IL-8 Release

Cell culture supernatants from monolayers exposed to TcdA 1 pg/mL–1 μg/mL were collected 24 h post-challenge to examine whether the toxin induces the release of IL-8 from the Caco-2 cells. However, none of the TcdA concentrations resulted in the release of IL-8 (Supplementary Figure S4).

### 2.4. Neither AOS nor COS Prevent TcdA-Induced Cell Cytotoxicity after 24 h

**Figure 2:**
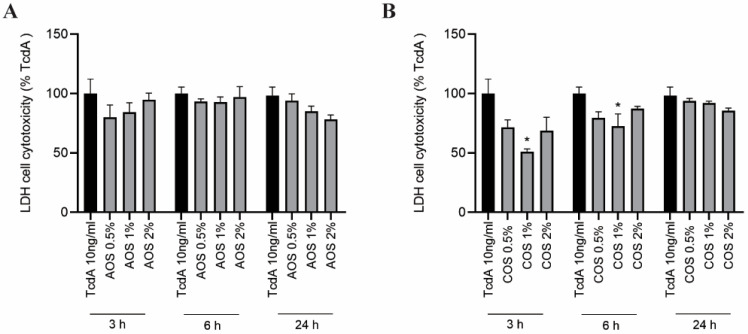
LDH cell cytotoxicity assay. Caco-2 monolayers were pre-treated with AOS or COS 0.5–2% for 24 h. Thereafter, cells were triggered with TcdA 10 ng/mL and a co-incubation period of a further 3, 6, and 24 h with either (**A**) AOS or (**B**) COS followed. TcdA-induced cell cytotoxicity was determined by measuring the LDH release to the cell culture supernatant. Results are expressed as a percentage of TcdA-treatment as mean ± SEM of three independent experiments, each performed in triplicate (* *p* ≤ 0.05: significantly different from the TcdA-challenged cells, as obtained using a one-way ANOVA test).

Caco-2 monolayers were pre-treated with increasing AOS or COS concentrations (0.5–2%), and after 24 h of incubation the cells were exposed to the TcdA cytotoxic concentration of 10 ng/mL and co-incubated with either AOS or COS for a further 3, 6, and 24 h. As depicted in Figure 2A, neither of the AOS concentrations significantly decreased LDH leakage compared to untreated TcdA-challenged cells throughout the 24 h of co-incubation. However, 24 h post-challenge, a concentration-dependent trend of cell cytotoxicity reduction was observed, with AOS 2% decreasing LDH levels by 20%. COS 1% exhibited cytoprotective activity, as demonstrated by the significantly diminished LDH leakage after 3 and 6 h of TcdA addition. This effect did not persist after 24 h. COS 0.5% and 2% did not exert any significant protective effect (Figure 2B).

### 2.5. TcdA Impairs Caco-2 Monolayer’s Integrity

**Figure 3:**
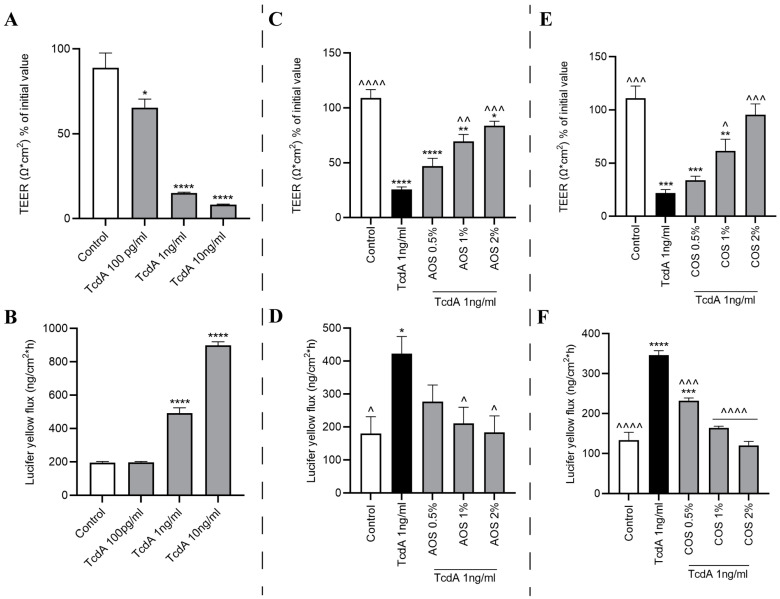
Effect of TcdA, AOS, and COS on epithelial barrier integrity: Caco-2 cells grown on Transwell^®^ inserts for 17–21 days were (**A**,**B**) treated with increasing TcdA concentrations for 24 h or pre-treated with increasing (**C**,**D**) AOS or (**E**,**F**) COS concentrations for 24 h and subsequently triggered with TcdA 1 ng/mL, with a further TcdA/NDO co-incubation of 24 h. TEER values were measured prior to and after the 24 h TcdA challenge. For the paracellular tracer flux assay, LY (20 μg/mL) was added apically after 24 h of TcdA exposure, and the amount of LY at the basolateral side was quantified after 5 h. Results are expressed as a percentage of initial value (TEER) or the amount of tracer transported [ng/(cm^2^ × h)] as mean ± SEM of three independent experiments, each performed in triplicate (* *p* ≤ 0.05, ** *p* ≤ 0.01, *** *p* ≤ 0.001, and **** *p* ≤ 0.0001: significantly different from the unstimulated cells; ^ *p* ≤ 0.05, ^^ *p* ≤ 0.01, ^^^ *p* ≤ 0.001, and ^^^^ *p* ≤ 0.0001: significantly different from the TcdA-stimulated cells, as obtained using a one-way ANOVA test.

The deleterious effects of TcdA on the integrity of Caco-2 monolayers were investigated by stimulating the cells with increasing TcdA concentrations (100 pg/mL–10 ng/mL) for 24 h, and TEER was measured after 3, 6, and 24 h post-challenge (Supplementary Figure S5A). The highest concentration (10 ng/mL) significantly dropped TEER already after 3 h, as well as at 6 and 24 h post-challenge, following a time-dependent trend. The non-cytotoxic concentrations of 1 ng/mL and 100 pg/mL TcdA induced significant TEER reductions after 24 h of toxin addition. Specifically, the effect of TcdA 1 ng/mL approached the levels of TcdA 10 ng/mL (*p* < 0.0001), while the lowest concentration of 100 pg/mL was less potent (*p* < 0.05) (Figure 3A). Similar results were obtained upon calculation of the total AUC, based on which the AUC for TcdA 1 ng/mL and 10 ng/mL were significantly lower compared to the control (Supplementary Figure S5B). To examine whether the TcdA-mediated disruption of TJs was reflected in an increased paracellular flux of impermeable tracers, the transportation of LY from the apical to the basolateral chamber was determined. After 5 h of LY addition to the apical compartment, both TcdA 1 ng/mL and 10 ng/mL resulted in a significant LY transport to the basolateral side, in accordance with the TEER results. By contrast, the lowest concentration did not affect the passage of LY (Figure 3B).

### 2.6. AOS and COS Possess Barrier-Protective Capability against TcdA-Induced Caco-2 Monolayer Disruption

Next, to evaluate the barrier-protective capacity of the NDOs against the noxious effects of TcdA on the epithelial monolayer, Caco-2 cells were pre-treated with AOS or COS (0.5–2%) for 24 h. Then, TcdA 1 ng/mL was added apically, a co-incubation period of a further 24 h followed, and TEER measurements and LY flux assay were performed as described in materials and methods. As illustrated in Figure 3C–F, both NDOs prevented TcdA-mediated TEER decrease and increased LY transport in a concentration-dependent manner. Even though AOS 1% and 2% significantly alleviated the TEER reduction compared to TcdA-stimulated cells, TEER levels were also significantly lower than those of unstimulated cells. A total of 2% AOS exerted the most potent effect, while the lowest concentration (0.5%) did not exert any protective effect (Figure 3C). Similarly, 1% and 2% AOS abolished the TcdA-induced increased flux in LY compared to TcdA-treated cells, while compared to control negative, pre-treatment with all of the AOS concentrations seemed to maintain the LY transportation at the levels of unstimulated cells (Figure 3D). As shown in Figure 3E, pre-treatment with COS 2% completely abolished the TcdA-induced TEER drop, as resistance values were significantly different from TcdA-challenged monolayers. COS 1% also mitigated TcdA-induced TEER decreases, presenting a significant difference compared to TcdA-challenged cells but also when compared to the control. By contrast, COS 0.5% failed to prevent TEER decreases induced by TcdA. With regards to LY flux, all of the COS concentrations were capable of significantly reducing LY transportation compared to TcdA-triggered monolayers; while compared to unstimulated cells, only COS 1% and 2% seemed to halt LY passage from the apical to the basolateral compartment. Finally, COS 0.5% failed to attenuate the increased tracer transport (Figure 3F) significantly. Then, the levels of ZO-1 and occludin were investigated via Western blotting to examine whether the observed effects on paracellular permeability were accompanied by alterations on TJ protein levels. Interestingly, TcdA 1 ng/mL did not induce any changes in either of these TJ proteins, and neither did the total (pre- and post-TcdA challenge) treatment with AOS or COS present any effect (Supplementary Figure S6A–D).

### 2.7. AOS and COS Prevent the TcdA-Induced Occludin Mislocalization

**Figure 4:**
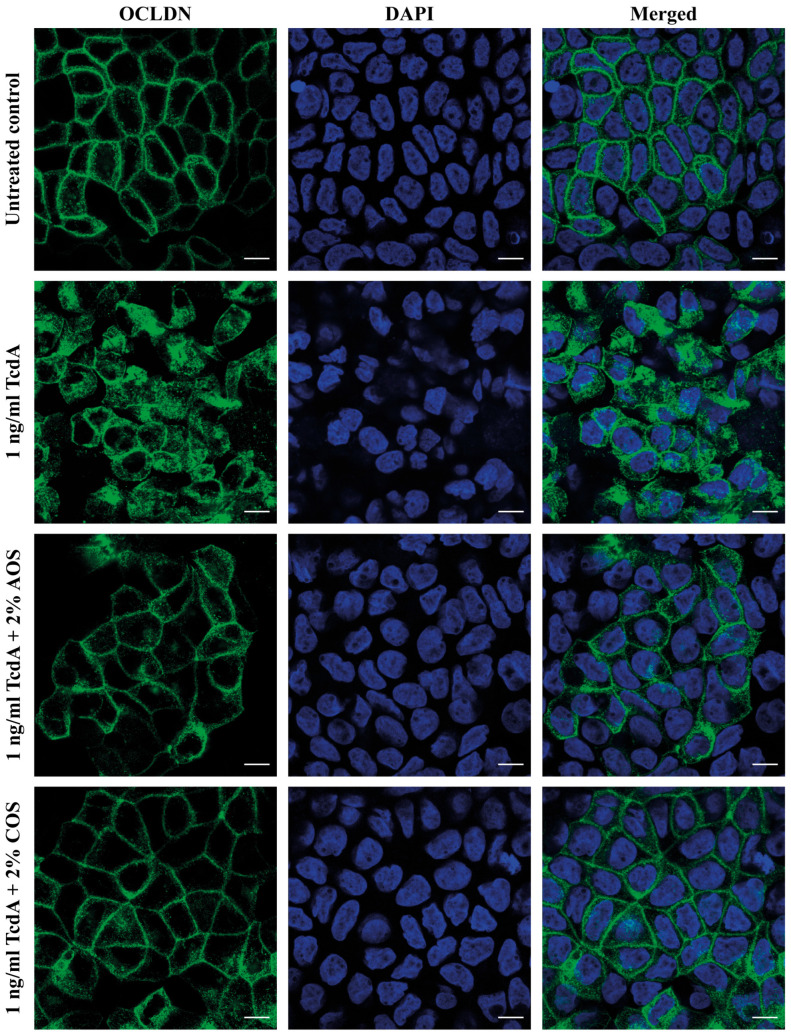
Immunofluorescence microscopy of occludin. Caco-2 monolayers grown on inserts were either pre-incubated with AOS 2% or COS 2% (24 h) and then exposed to TcdA 1 ng/mL (24 h) or exposed solely to TcdA 1 ng/mL (24 h), and the localization of occludin was studied via immunofluorescence staining (green). Nuclei were stained with Hoechst (blue). Untreated cells served as the negative control. Scale bar: 10 μm.

Considering the absence of any TcdA-mediated effect on the TJ protein levels, the localization of occludin was investigated by confocal microscopy. Caco-2 monolayers were first treated with AOS or COS 2% for 24 h. Thereafter, cells were co-incubated with TcdA 1 ng/mL and AOS 2% or COS 2% for another 24 h. Then, cells were fixed for immunostaining and fluorescence microscopy of occludin. Indeed, stimulation of the cells with TcdA solely resulted in an altered distribution pattern of the TJ protein compared with the untreated control (Figure 4). However, when the monolayers were pre-incubated with either AOS or COS, occludin appeared with a more distinct pattern, similar to the control, signifying that these NDOs can mitigate or even abrogate the TcdA-induced alterations in occludiN′s localization. In Supplementary Figure S7 the corresponding isotype and negative control for this immunofluorescence staining of occludin are depicted.

### 2.8. Both NDOs Facilitate the Reassembly of TJs after Calcium Deprivation in Caco-2 Cells

**Figure 5:**
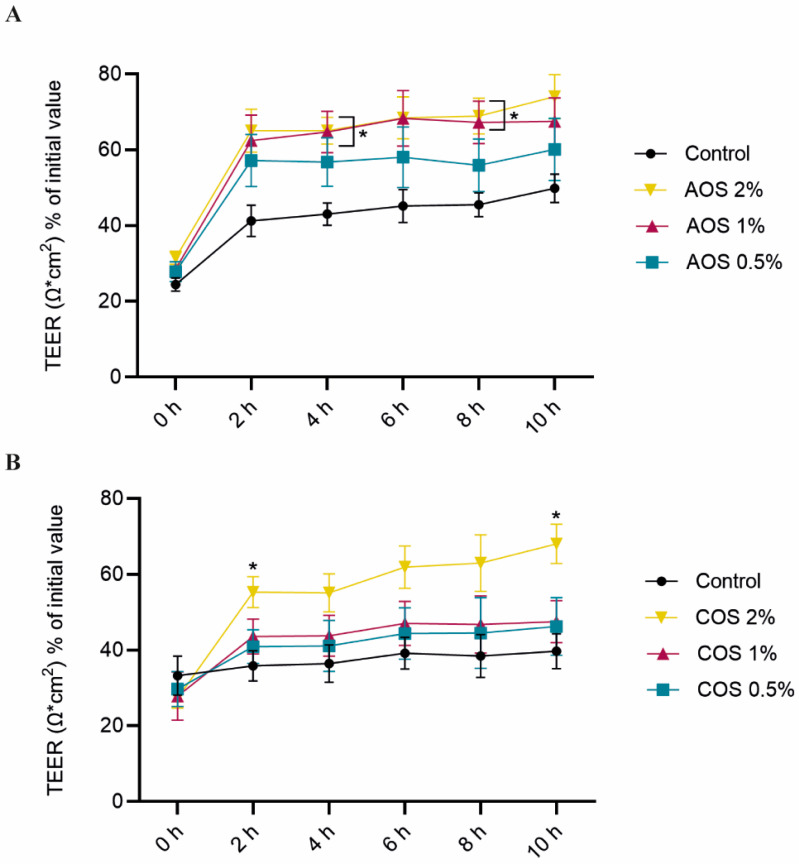
Calcium switch assay for AOS and COS. To examine whether the herein studied NDOs accelerate TJ reassembly, Caco-2 cells grown on Transwell^®^ inserts were pre-treated with increasing AOS/COS concentrations (24 h) prior to transient calcium deprivation with HBSS-EGTA to disrupt intercellular contacts. TEER values were measured during recovery (at 0, 2, 4, 6, 8, and 10 h) in complete, calcium-containing DMEM supplemented with either (**A**) AOS or (**B**) COS. Results are expressed as a percentage of the initial value as mean ± SEM of three independent experiments, each performed in triplicate (* *p* ≤ 0.05: significantly different from the unstimulated cells, as obtained using a one-way ANOVA test for each time point separately).

A calcium switch assay was conducted to examine whether AOS and COS can accelerate the reassembly of intercellular junction complexes under dynamic disassembly/reassembly conditions. Compared to the unstimulated cells, AOS 2% facilitated the re-establishment of cell–cell contacts 4 and 8 h after calcium recovery. AOS 1% also enhanced the TJ reassembly during 4 and 8 h, while AOS 0.5% did not exert any significant effect compared to the control (Figure 5A). In addition, the corresponding total AUC for AOS 2% was significantly different compared to the control, contrary to the lowest concentrations (Supplementary Figure S8A). COS 2% stimulated TJ reassembly after 2 and 10 h of calcium replenishment. The lower concentrations (1% and 0.5%) did not significantly affect TEER restoration (Figure 5B). Finally, calculation of the total AUC indicated that only COS 2% tended to exert a significant effect (P:0.0575) (Supplementary Figure S8B).

## 3. Discussion

CDI is a leading cause of nosocomial infection, which initially mainly occurred in developed countries but is now emerging as a health-related global threat. The development of antibiotic resistance and the emergence of hypervirulent strains are considered drivers of CDI outbreaks with significantly high mortality rates [6,7]. CDI is caused by the opportunistic bacterium *C. difficile,* whose pathogenetic effects are mainly caused by its exotoxins. TcdA is a highly potent enterotoxin that is the major cause of *C. difficile* enterotoxicity via the induction of both cytopathic and cytotoxic effects and is a well-established disruptor of epithelial TJs [50]. TJs of the intestinal epithelium constitute a key element of the intestinal barrier as these intercellular protein complexes seal the paracellular space between neighboring cells and form a continuous intercellular barrier across the interspace of epithelial cells. Impaired TJ functionality, followed by permeation of luminal contents, induces inflammatory and immune responses, ultimately triggering the leaky gut syndrome [12]. The primary target of TcdA is the inactivation of RhoA, a crucial regulator of the actin cytoskeleton and TJ assembly. Inactivation of this small GTPase by glycosylation results in alteration of the cellular structure and impaired TJ integrity. Subsequently, the increased epithelial permeability triggers acute inflammatory and immune responses that further contribute to the leaky gut and excessive secretion of ions. NDOs are prebiotics able to selectively stimulate the growth of health-promoting bacteria and, via their fermentation products, exert numerous beneficial effects for the host activities, including the preservation of a well-functioning mucosal barrier and a homeostatic immune system. Apart from their microbiota-related beneficial activity, emerging evidence demonstrates that numerous NDOs own direct barrier-protective properties, i.e., via mechanisms independent of the gut flora, including direct induction of TJ signaling and protection from various TJ-disrupting triggers such as pathogens, toxins, inflammatory-inducing cytokines, and LPS [17]. Moreover, numerous NDO categories have been shown to demonstrate antibacterial activity via inhibition of bacterial growth, adhesion, and biofilm formation, among others [21,30]. Among these oligosaccharides, AOS and COS seem very promising due to their versatile properties, though their direct antimicrobial and barrier-reinforcing abilities have not been widely investigated. Here, we addressed the hypothesis that AOS and COS can affect the growth of *C. difficile* as intact compounds and exert direct barrier protection against TcdA-mediated disruption of the epithelial layer.

Initially, the potential antibacterial activity of AOS and COS against *C. difficile* was investigated. Previous studies have revealed that both NDOs reduce the growth of other exotoxin-producing bacteria in vitro. AOS significantly decreased the growth of *Streptococcus agalactiae* [42] and *E. coli* [41]. COS inhibited the growth of *E. coli* [46,47,49], *Bacillus cereus* [49,51,52], and *S. aureus* [46,52,53]. Here, based on the MIC assay, the MIC for AOS was 2% and for COS it was 1%, a significantly diminished bacterial growth was already observed at 0.25% for both compounds. This is the first time that the antibacterial effects of AOS and COS have been examined against *C. difficile*. Interestingly, a recent study demonstrated that FOS, another well-established group of prebiotic oligosaccharides, exerted antibacterial effects against *C. difficile* by inhibiting biofilm formation and cell adhesion [54]. These three NDOs suppress *C. difficile* growth despite their structural differences and, consequently, distinct physicochemical characteristics that affect cell surface receptor or other intermolecular interactions. Hence, they might also have distinct mechanisms of action underlying the antibacterial activity on *C. difficile*. FOS are composed of D-fructose monomers usually with β-(2 → 1) linkages and a terminal α-(1 → 2) linked D-glucose residue and are uncharged in normal pH. The main components of AOS are 1,4-linked β-D-mannuronic acid and 1,4-linked α-L-guluronic acid and of COS 1,4-linked GlcNAc and 1,4-linked GlcN, which carry negative and positive charges, respectively [21]. Considering the multiple antimicrobial effects of both AOS and COS against numerous Gram + and Gram − bacteria, including motility and QS-signaling inhibition, and pathogenic membrane disruption, further studies regarding the anti-pathogenic effects of AOS and COS against *C. difficile*, and the investigation of structure–function relationships, seem very promising.

TcdA mediates cell cytotoxicity by inducing inflammatory responses, oxidative stress, and programmed cell death. Previous studies on IECs have demonstrated that both NDOs have anti-apoptotic potential in vitro. AOS reduced TNF-α-induced apoptotic rates in IPEC-2 monolayers via inhibition of the cell surface death receptor TNFR1-initiated caspase-8-mediated pathway, thus halting the extrinsic apoptotic pathway [20]. In another study, AOS inhibited LPS from binding to TLR-4 receptors of IPEC-2 cells and suppressed TLR-4/NF-κB-mediated apoptosis [23]. Similarly, COS prevented LPS from binding to T84 cells and reduced TNF-α- and oxidative stress-induced apoptosis [27]. In this study, we investigated the protective abilities of AOS and COS against the TcdA-induced cytotoxic and cytopathic effects. Based on the LDH leakage assay, pre-treatment with AOS or COS (0.5–2%, 24 h), and following co-incubation with AOS or COS and 10 ng/mL TcdA (cytotoxic concentration), failed to mitigate the toxin-induced cytotoxicity 24 h post-challenge. However, both NDOs presented a cytotoxicity-decreasing tendency at 24 h. In addition, COS 1% exhibited a cytoprotective effect at 3 and 6 h post-challenge. Taken together, these results show that AOS and COS do not effectively protect Caco-2 cells from TcdA cytotoxicity but might have a slight cytoprotective potential. Interestingly, the ELISA assay showed that none of the toxin concentrations (TcdA 1 pg/mL–1 μg/mL) managed to elicit IL-8 release. This is per previous studies demonstrating the impotence of TcdA in this concentration range on causing IL-8 production by Caco-2 monolayers [55,56]. By contrast, in other studies using HT-29 and T84 monolayers, TcdA mediated the release of IL-8 [56,57], while in a study on Caco-2 cells, TcdA managed to induce TNF-α and IL-6 release [11]. Even though TcdA is a well-recognized inflammatory enterotoxin, little is known regarding the underlying mechanisms of innate immune system activation and stimulation of pro-inflammatory cytokine release [58]. Further studies are necessary to elucidate under which conditions TcdA elicits an inflammatory response, and to determine the implicated cellular pathways.

Next, Caco-2 monolayers were grown on Transwell^®^ inserts to investigate the use of AOS and COS in protecting against TcdA-mediated disruption of epithelial barrier function. The integrity of the monolayer was assessed by measurement of TEER and apical to basolateral flux of LY. TEER measurements are a standard technical approach to evaluate barrier properties and dynamics in vitro and indicate the ionic conductance of the paracellular pathway [59]. Transport studies using nonelectrolyte tracers indicate the TJ pore size and the paracellular water flow [60]. Numerous studies have demonstrated the TcdA-induced cytopathic effects, principally involving disruption of intercellular cell–cell junctions, thus increasing epithelial permeability in vitro [7,11,15,50]. Exposure of the Caco-2 monolayers to TcdA 100 pg/mL, 1 ng/mL (non-toxic concentrations 24 h post-exposure), and 10 ng/mL (toxic concentration 24 h post-exposure) impaired the monolayer’s integrity in a time- and concentration-dependent manner. TcdA 10 ng/mL induced a significant TEER drop already at the third hour of incubation, in accordance with the LDH assay results, which demonstrated that the TcdA-induced cytotoxicity at this concentration is evident already 3 h post-exposure. On the other hand, 100 pg/mL, and 1 ng/mL TcdA managed to significantly decrease TEER only after an incubation period of 24 h. Moreover, in agreement with the TEER results, challenge with 1 and 10 ng/mL TcdA for 24 h resulted in significant transportation of LY from the apical to the basolateral compartment, while LY flux for TcdA 100 pg/mL was comparable with levels of unstimulated cells. Hence, considering that 100 pg/mL and 1 ng/mL TcdA do not induce cell death when added to Caco-2 monolayers for 24 h, while 10 ng/mL TcdA does, we can claim that TcdA 100 pg/mL and especially TcdA 1 ng/mL exerted cytopathic effects which led to increased paracellular permeability, while the acute TEER drop induced by TcdA 10 ng/mL was due to its cytotoxicity.

Here, we sought to investigate the protective potential of AOS and COS against the TcdA-mediated cytopathic effects, which are reflected by an increased paracellular permeability. Hence, to examine the protective abilities of these NDOs regarding the monolayer’s integrity, the selection of a non-cytotoxic TcdA concentration was requisite. Having established TcdA 1 ng/mL as a cytopathic concentration based on both TEER and LY transport assays, the barrier-protective potential of AOS and COS was examined. Both NDOs have been previously shown to confer barrier protection in vitro against various stimuli known for disrupting epithelial TJs. AOS reinforced the epithelial integrity of IPEC/J2 monolayers in a mannose receptor (MR)-dependent manner [38,61] and saved the monolayer’s integrity from the TNFα [20], LPS [23] challenges via alleviating inflammation. COS have also been shown to promote TJ integrity via an 5′ AMP-activated protein kinase (AMPK)-mediated acceleration of TJ reassembly in T84 monolayers [39]. Moreover, COS prevented barrier impairments of LPS-challenged T84 and IPEC-J2 cells [27,40], of TNF-a-challenged IPEC-J2 cells [62], and of DSS-challenged Caco-2 cells [32], by suppressing inflammatory responses. In this study, both AOS and COS succeeded in protecting the integrity of the monolayer concentration-dependently. The highest COS and AOS concentrations (1–2%) abrogated and alleviated, respectively, the TcdA-induced TEER drop. Both NDOs (1–2%) completely prevented the flux of LY from the apical to the basolateral chamber. To investigate whether changes in TJ protein levels accompany the observed effects, a Western blot analysis was performed. Strikingly, despite the evident TcdA-mediated increased paracellular permeability, no alterations at the protein expression of the crucial TJ proteins, ZO-1 and occludin, were observed according to the Western blot assay. In previous studies using Caco-2 cells, TcdA challenge altered ZO-1 and occludin protein expression and localization; though in all of these studies, the TcdA concentration applied was cytotoxic [11,50,63]. TcdA has a time- and concentration-dependent toxicity, thus a plausible explanation for the absence of TJ protein level changes is that the concentration of 1 ng/mL was not capable of suppressing TJ protein expression within this time frame. Furthermore, members of the claudin family of TJ complexes might also be associated with the observed impact of TcdA [64]; however, additional research is required to confirm this connection. Previous studies have demonstrated that the TcdA-mediated increased paracellular permeability is not necessarily the aftermath of actin cytoskeleton depolymerization and that the early increases in TJ permeability are mediated by signaling events, including activation of protein kinase C isoforms α/β (PKCα/PKCβ) [65,66]. The PKC family of isozymes are involved in the regulation of TJs via direct phosphorylation of TJ proteins including ZOs and occludin, and the conventional PKCs (α, β1, β2, and γ) have been associated with TJ disassembly, TEER decreases, and TJ mislocalization [67]. Moreover, in a study using T84 monolayers, TcdA impaired the monolayer’s integrity as indicated by TEER and tracer flux assays, though without affecting ZO-1 and occludin protein levels. In the same study, confocal microscopy showed that TcdA induced displacement of TJ proteins (ZO-1, -2, and occludin) from the lateral membrane of TJs and F-actin disorganization. These effects were caused by the TcdA-mediated decreased association of the cytoplasmic plaque protein ZO-1 with the actin cytoskeleton, and this event was accompanied by a decreased pool of occludin and its internalization from the lateral TJ membrane since the functions of this TJ protein require binding to ZO proteins. Hence, the TcdA-mediated increase in paracellular permeability was caused by the disassembly of TJs rather than being due to changes in TJ protein abundance [68,69]. Thus, it is possible that the TcdA concentration used here, even though not cytotoxic, managed to impair the integrity of the Caco-2 monolayers via mislocalization of TJ proteins, but without altering their expression, as an early cellular response to TcdA. Accordingly, in the present study, the intoxication of Caco-2 monolayers with TcdA 1 ng/mL resulted in disturbed occludin architecture as observed by immunofluorescence microscopy. Notably, when the monolayers were pre-treated with either AOS 2% or COS 2%, the TcdA-induced displacement of the TJ protein was prevented, further supporting the barrier-protective capability of these NDOs against the toxin A of *C. difficile*.

Next, to explore the barrier-reinforcing properties of AOS and COS, a calcium switch assay was performed. This assay is based on the principle that intercellular TJs are disrupted upon deprivation of extracellular Ca^2+^, leading to a marked TEER decrease. Once Ca^2+^ is replenished, TJs are reassembled to the membrane periphery close to the apical surface [70]. As mentioned above, COS have been previously shown to accelerate TJ reassembly in T84 monolayers via a CaSR-Gq-PLC-IP3-CaMKKβ-dependent pathway that leads to AMPK stimulation [39]. However, AMPK stimulation is not cell-line specific, as COS also induced an increase in pAMPK levels in Caco-2 and HT-29 monolayers [39]. Notably, FOS also promoted TJ dynamics via induction of intracellular Ca^2+^ signaling and acceleration of TJ reassembly, following the exact mechanism as COS in T84 monolayers [71]. In the present study, pre-treatment of Caco-2 monolayers with 2% COS promoted the reassembly of the TJ complex, with significant differences from the control group already after 2 h of incubation, which also persisted after 10 h. Moreover, AOS also presented this potential since 1% and 2% AOS significantly increased TEER compared to unstimulated cells at various time points, though the highest concentration was more effective. Based on a study using Caco-2 cells, the TJ reassembly process depends on the activation of AMPK, a crucial TJ assembly/disassembly regulator, and that AMPK stimulation ensures a better recovery of epithelial barrier function after injury [72]. Thus, like FOS and COS, AOS seems to stimulate AMPK signaling via interactions with a Ca^2+^ sensing receptor (CaSR) of the epithelial cell surface. Considering the importance of AMPK in the facilitation of TJ recovery, we speculate that the barrier-protective effects against TcdA-induced epithelial damage are, at least in part, mediated by stimulation of the AMPK signaling. Furthermore, it is well-established that the receptor binding domain (B Domain) of TcdA interacts with carbohydrate structures containing galactose (Gal)- and GlcNAc found in the epithelial cell surface [5]. Previous studies demonstrated that such a receptor is the trisaccharide Gal-α-(1,3)-Gal-β-(1,4)-GlcNAc present in the intestine of infant hamsters, but not expressed in humans [73,74]. Nonetheless, it has been revealed that TcdA binds to carbohydrate antigens designated X, I, and Y, which are existent on the intestinal epithelium of humans and have a type 2 core Gal-β-(1,4)-GlcNAc, with the NAc part being crucial for toxin binding. However, TcdA contains multiple binding sites for carbohydrate ligands [75]. Hence, another plausible mechanism underlying the AOS and COS-mediated protection against TcdA toxicity, may involve antagonism for the glycoconjugate receptors of the host epithelial cell membranes targeted by TcdA. In addition, the possibility of TcdA inactivation upon interaction with AOS or COS should be investigated in future studies by removing the NDOs prior to TcdA addition. Alternatively, Caco-2 monolayers could be exposed to a pre-mixed cocktail of AOS or COS and TcdA to clarify whether some of the protective effects could result from direct interaction between the NDOs and TcdA. Furthermore, considering that the main components of COS are 1,4-linked GlcNAc and 1,4-linked GlcN, it is highly likely that the superiority of this NDO over AOS, regarding the protection against TcdA toxicity, is directly linked to the presence of the GlcNAc monomers. Finally, TcdB is also a major virulence factor of *C. difficile*, which based on accumulating evidence, plays a key role in the pathogenesis of both localized (i.e., intestinal) and systemic CDI. Future studies might unravel a cytoprotective potential of AOS and COS against TcdB alone and in combination with TcdA and shed light on the underlying mechanisms of action.

## 4. Conclusions

The objective of this study was to evaluate the protective effects of AOS and COS against a major causative pathogen of hospital-acquired infection, the bacterium *C. difficile,* as well as against the deleterious effects of its principal enterotoxin, TcdA. Based on our findings, AOS and COS 0.25–4% significantly reduced bacterial growth following a concentration-dependent trend. Nevertheless, neither AOS nor COS were successful regarding the TcdA-mediated cytotoxicity 24 h post-challenge. Furthermore, based on the barrier integrity assays and confocal microscopy, both AOS and COS protected the epithelial barrier from TcdA-mediated disruption. Another interesting finding was that both NDOs accelerated the reassembly of epithelial TJs of Caco-2 cells and thus can strengthen the intestinal barrier via promoting the sealing of the paracellular route. Taken together, our findings uncover two very promising agents, AOS and COS, against *C. difficile* bacteria and TcdA, opening the route for further studies aiming to alternative therapeutic strategies against CDI, while in parallel revealing the cytoprotective and barrier-enhancing abilities of these NDOs. Further studies are warranted, though, to unravel the underlying mechanisms by which AOS and COS exert their health-beneficial effects.

## 5. Materials and Methods

### 5.1. Bacterial Strains and Culture Conditions

*Clostridioides difficile* (ATCC 9689) was grown in brain heart infusion (BHI) medium-containing plates (Biotrading, Mijdrecht, The Netherlands) for 24 h at 37 °C under anaerobic conditions in a vinyl anaerobic chamber (Coy labs) containing a gas mixture of 85% N_2_, 10% CO_2,_ and 5% H_2._ Colonies of *C. difficile* were checked by Gram-staining [76] and by matrix-assisted laser desorption ionization-time of flight mass spectrometry (MALDI-TOF MS) (Bruker, Delft, The Netherlands) as described by Wieser et al. (2012) [77]. Thereafter, single colonies were inoculated in BHI broth and incubated overnight at 37 °C. After incubation, the optical density (OD) of the bacterial culture was measured (OD_600_), and the bacterial density was adjusted to OD_600_ = 0.2 in BHI to be used in MIC assays.

### 5.2. Cell Culture

Colorectal adenocarcinoma (Caco-2) cells were purchased from the American Type Culture Collection (Code HTB-37) (Manassas, VA, USA, passages 27–45) and were cultured in Dulbecco’s modified Eagle medium (DMEM, code 42430025), supplemented with 10% (*v*/*v*) inactivated fetal calf serum (FCS), 1% (*v/v*) L-glutamine, 1% (*v/v*) non-essential amino acids, and penicillin (10,000 U/mL)/streptomycin (10,000 μg/mL). Cells were maintained in vented 75 cm^2^ flasks in a humidified cell culture incubator with 5% CO_2_ at 37 °C. Confluent cells (90%) were washed two times with phosphate-buffered saline (PBS) and detached with 0.05% trypsin/0.54 mM ethylene diamine tetraacetic acid (EDTA). Caco-2 cells were seeded at a density of 3 × 10^4^/well in 96-well plates and were grown for 7 days (37 °C, 5% CO_2_) until a confluent monolayer was achieved. The medium was refreshed every other day.

### 5.3. Oligosaccharides and TcdA

COS (purity > 90%) and AOS (purity > 85%) were purchased from BZ Oligo Biotech Co., Ltd. (Qingdao, Shandong, China). COS originated from marine biological sources (shrimp and crab shells), and AOS were produced by the degradation of algin. The stock solutions of all NDOs were freshly prepared by dissolving them in BHI (minimum inhibitory concentration assay) or DMEM before each experiment, and the pH of the solution was adjusted to 7.2–7.4, and finally, the treatments were microfiltered using a syringe filter (0.2 μm, Corning, Rockville, MD, USA). *C. difficile* toxin A (TcdA) was purchased from Sigma-Aldrich (St. Louis, MO, USA) and reconstituted in 250 µL sterile dH_2_O to obtain the stock solution. For each experiment, the different concentrations of TcdA were freshly prepared by diluting the stock solution with DMEM.

### 5.4. Determination of the Minimum Inhibitory Concentration (MIC)

The antibacterial capacity of AOS and COS against *C. difficile* was determined by analyzing the MIC as previously described [41,78]. NDOs were serially diluted in 96-well U-bottom polypropylene plates (Corning Costar, Cambridge, MA, USA) until reaching the final volume of 100 μL. Subsequently, 100 μL of bacterial inoculums (*C. difficile*) with OD_600_ = 0.2 (approximately 4.10^7^ colony-forming units [CFU]/mL) were added to the serially diluted NDOs [79,80]. Starting OD was measured at 600 nm with a FLUOstar Omega microplate reader (BMG Labtech GmbH, Ortenberg, Germany). The plates were incubated anaerobically in a vinyl anaerobic chamber (Coy labs) overnight at 37 °C. Then, 100 μL of culture medium was transferred to 96-well F-bottom polystyrene microtiter plates (Corning Costar, Cambridge, MA, USA), and the signal was measured at 600 nm with a FLUOstar Omega microplate reader (BMG Labtech, Ortenberg, Germany). Bacteria growth in BHIs without treatment served as a positive control, and BHIs containing different concentrations of COS or AOS were used as a negative control. The starting OD was subtracted from the final OD. Finally, the MIC was considered as the lowest concentration that inhibits bacterial growth in comparison to the positive control groups [42].

### 5.5. Cell Viability Assays

Cell viability was examined using two different colorimetric assays. First, the 3-(4,5-Dimethylthiazol-2-Yl)-2,5-Diphenyltetrazolium Bromide (MTT) colorimetric assay (Sigma-Aldrich, St. Louis, Mo, USA) was performed to determine cell viability of the monolayers after stimulation with either AOS, COS, or TcdA. Briefly, Caco-2 cells were grown on 96-well plates, and the confluent monolayers were exposed to different concentrations of NDOs (0.125–4%) or TcdA (1 pg/mL–1 μg/mL). After 48 and 24 h, for the NDOs and TcdA, respectively, 20 μL MTT working solution (5 mg/mL in PBS) was added to 100 µL of culture medium. Following 2 h of incubation, the supernatant was removed, cells were lysed with DMSO, and the absorbance was measured at 600 nm using an iMark microplate reader (BioRad, Hercules, CA, USA). The viability rate of the Caco-2 cells was calculated based on the following equation: (mean absorbance of treatment cells/mean absorbance of control cells) × 100.

Secondly, the lactate dehydrogenase (LDH) assay was conducted (Promega, Madison, WI, USA). The release of LDH is a marker of cell membrane integrity; thus, measuring the levels of LDH in the culture supernatant is a stable index of cellular injury extent. Caco-2 cells were grown on 96-well plates, and upon reaching confluency, the monolayers were pre-treated with the NDOs (0.5–2%) for 24 h. Then, the cells were triggered with either TcdA 10 ng/mL alone or in addition to AOS/COS for a further 24 h. The supernatant was collected after 3, 6, and 24 h, and LDH leakage into the supernatant was determined using a colorimetric reaction according to the manufacturer’s protocol. The absorbance was read at 560 nm using an iMark microplate reader (BioRad).

### 5.6. ELISA Assay for IL-8 Secretion

Culture supernatants from the MTT assay for TcdA (1 pg/mL–1 μg/mL) were collected 24 h post-exposure to quantify the inflammatory marker IL-8 via an enzyme-linked immunosorbent assay (ELISA). A corresponding IL-8 kit (Invitrogen, Carlsbad, CA, USA) was used according to the manufacturer’s instructions.

### 5.7. Transepithelial Electrical Resistance (TEER) Measurement

Caco-2 cells were seeded on 0.33 cm^2^ high pore density polyethylene terephthalate membrane Transwell^®^ inserts with 0.4 μm pores (Corning Costar Corp., New York, NY, USA) placed in a 24-well plate at a density of 0.1 × 10^5^ cells/insert. The medium was refreshed every other day. Barrier integrity assays were started after obtaining a confluent monolayer at day 17–20 of culturing with TEER values in the range of ±700 Ω·cm^2^. To select one TcdA concentration for the subsequent experiments, cells were exposed apically to 100 ng/mL, 10 ng/mL, and 1 μg/mL TcdA and incubated for 24 h. Having determined TcdA 1 ng/mL as the optimal concentration, the cells were initially pre-treated with AOS/COS 0.5–2% for 24 h. Then, the cells were apically challenged with TcdA (1 ng/mL) alone or in addition to AOS/COS for a further 24 h. The integrity of the monolayer was determined prior to and after TcdA exposure (3, 6, and 24 h) by measuring TEER levels using a Millicell-ERS volt-ohmmeter (Millipore, Temecula, CA, USA). Average TEER values for untreated cell monolayers were in the range of 700 ± 20 Ω·cm^2^. The results are expressed as a percentage of the initial value.

### 5.8. Paracellular Tracer Flux Assay

Paracellular permeability across the Caco-2 cell monolayer was determined by measuring the flux of the membrane-impermeable molecule lucifer yellow (LY). The transportation studies from the apical to the basolateral side were performed with 20 μg/mL of LY (molecular weight: 0.457 kDa, Sigma Chemical Co., Ltd., St Louis, MO, USA), which was added to the apical compartment (200 μL) of the inserts 24 h post-TcdA exposure. Medium from the basolateral chamber was collected 5 h after the tracer’s addition. The amount of LY in the basolateral compartment was determined by measuring the fluorescence intensity at excitation and emission wavelengths of 405 and 500–550 nm, respectively.

### 5.9. Calcium Switch Assay

Caco-2 cells grown on inserts were pre-treated with 0.5–2% AOS/COS, which were added to the apical compartment for 24 h, and TEER values were measured. Subsequently, cells were washed with PBS, and the TJ protein complex was disrupted by incubation with 2 mM ethylene glycol-bis(2-aminoethyl ether)N,N,N′,N′-tetraacetic acid (EGTA, Sigma Chemical Co., Ltd., St Louis, MO, USA) in calcium- and magnesium-free Hank’s balanced salt solution (HBSS, Gibco, Invitrogen, CA, USA) for 20 min. Subsequently, the HBSS-EGTA was removed, cells were rinsed with PBS, and cell–cell contacts were allowed to re-establish by incubation with either complete cell culture DMEM (containing 2 mM CaCl_2_) or in DMEM supplemented with the different AOS/COS concentrations. TEER values were measured during this recovery period to examine the TJ reassembly progress every 2 h and for a time period of 10 h. The results are expressed as a percentage of the initial value.

### 5.10. Western Blot Analysis

Caco-2 monolayers grown on inserts were pre-treated with or without AOS or COS (0.5–2%) for 24 h and exposed to TcdA 1 ng/mL for a further 24 h. Thereafter, cells were lysed using 50 µL RIPA lysis buffer/well (Thermo Scientific, Rockford, IL, USA) containing protease inhibitors (Roche Applied Science, Penzberg, Germany). Next, cells were washed with cold PBS, and cells were lysed with 50 μL RIPA lysis buffer containing protease inhibitors (Roche Applied Science, Penzberg, Germany). After 30 min incubation with RIPA buffer, cells were harvested and centrifuged at 16.000 g for 20 min to yield a clear lysate. The lysates were normalized for protein content, and for total protein concentration assessment, a BCA protein assay kit (Thermo Scientific) was used. Equal protein amounts of heat-denaturated nonreduced samples were separated by electrophoresis (Criterion™ Gel, 4–20% Tris–HCl, Bio-Rad Laboratories Inc.) and blotted onto polyvinylidene difluoride membranes (Bio-Rad, Veenendaal, The Netherlands). Thereafter, membranes were blocked with PBS containing 0.05% (*v/v*) Tween-20 (PBST) and 5% (*w*/*v*) milk proteins for 2 h at room temperature. Subsequently, the membranes were incubated overnight at 4 °C with primary antibodies against ZO-1, occludin (1:1000, Invitrogen, Carlsbad, CA, USA), and monoclonal rabbit anti-human β-actin antibody (1:2000, Cell Signaling, Danvers, MA, USA) for equality of sample loading. Following washing with PBST, blots were incubated with appropriate horseradish peroxidase-conjugated secondary antibodies (1:2000, Dako, Glostrup, Denmark) for 1 h at room temperature. Next, membranes were washed in PBST, incubated in commercial ECL reagents (Amersham Biosciences, Roosendaal, The Netherlands), and finally exposed to X-ray film (Thermo Scientific, Antwerp, Belgium). The ChemiDoc™ MP imager (Bio-Rad Laboratories Inc.) was used to obtain the digital images, and signal intensities were quantified via the ImageJ 1.47 software and expressed as relative protein expression (optical density normalized with β-actin).

### 5.11. Immunofluorescence Microscopy of Occludin

Caco-2 monolayers grown on inserts were pre-treated with either AOS 2% or COS 2% for 24 h and then were intoxicated with TcdA 1 ng/mL for a further 24 h. Cells were fixated with 4% PFA/PBS solution (apically and basolaterally) for 10 min, washed twice with PBS, and permeabilized with Triton-X 100 (0.2% in PBS) for 15 min, followed by three washes with PBS. Next, cells were incubated with blocking solution (1% BSA, 5% NGS in PBS) for 1.5 h. Primary antibodies were diluted in 1% BSA in PBS, and cells were incubated with the primary antibody solutions overnight at 4 °C. For immunostaining against occludin, cells were incubated with a monoclonal rabbit anti-occludin antibody (Invitrogen 404700, 1:50), and the polyclonal rabbit IgG (Abcam ab 176094, 1:100, Abcam, Cambridge, UK) was used as an isotype control. The following day, cells were washed three times with PBS for 2 min per wash. The secondary antibody Alexa Fluor goat-anti rabbit 488 was diluted in 1% BSA in PBS (Invitrogen, 1:500), and cells were incubated with secondary antibody solution for 2 h in the dark. Nuclei were stained with DAPI (Sigma-Aldrich, 1:1000 in PBS/1% BSA) for 15 min in the dark followed by three washes with PBS for 2 min per wash. Thereafter, the filters were cut out of the inserts and mounted to slides using fluorescence mounting medium (Dako, Glostrup, Denmark). Thereafter, the slides were left to dry overnight in the dark and kept at 4 °C until imaging. All procedures were performed at room temperature unless stated otherwise. Immunofluorescence microscopy images were obtained using either a Leica TCS SP8 X confocal microscope (60× magnification) or a Zeiss LSM700 confocal microscope (63× magnification). Raw images were processed with ImageJ software.

### 5.12. Statistical Analysis

Data were reported as mean values ± SEM of at least three independent experiments (*n* = 3) routinely performed in triplicate (three wells/condition). Results were analyzed using Prism 8.0 GraphPad Software (GraphPad, San Diego, CA, USA). Statistical significance was determined using an ANOVA followed by a Dunnett post hoc test. Differences were considered statistically significant when *p* < 0.05.

## Data Availability

The data presented in this study are available on request from the corresponding author.

## Supplementary Materials

The following supporting information can be downloaded at: https://www.mdpi.com/article/10.3390/toxins15100586/s1, Figure S1: MTT cell viability assay; Figure S2: ELISA assay for IL-8; Figure S3: TEER trend and AUC of Caco-2 cells exposed to TcdA; Figure S4: Relative TJ protein expression in response to TcdA and TcdA/NDOs; Figure S5: Isotype and negative controls for immunofluorescence staining; Figure S6: Total AUC for calcium switch assay upon AOS/COS treatment; Figure S7: Isotype and negative controls for immunofluorescence staining; Figure S8: Total AUC for calcium switch assay upon AOS/COS treatment.

## Abbreviations

AMPK	5′ AMP-activated protein kinase
AOS	Alginate oligosaccharides
CaMKKβ	Calcium/Calmodulin-dependent protein kinase kinase β
CaSR	Calcium-sensing receptor
CDI	*C. difficile* infection
CDT	*C. difficile* transferase
COS	Chitin/chitosan oligosaccharides
DA	Degree of acetylation
DD	D Domain
DP	Degree of polymerization
DSS	Dextran sulfate sodium
*E. coli*	*Escherichia coli*
ELISA	Enzyme-linked immunosorbent assay
Gal	Galactose
GlcN	D-glucosamine
GlcNAc	N-acetyl-D-glucosamine
GTD	N-terminal glucosyltransferase domain
IP3	D-myo-inositol 1,4,5-trisphosphate
LDH	Lactate dehydrogenase
LPS	Lipopolysaccharide
LY	Lucifer yellow
MIC	Minimum inhibitory concentration
MTT	3-(4,5-Dimethylthiazol-2-Yl)-2,5-Diphenyltetrazolium Bromide
MW	Molecular weight
NDOs	Non-digestible oligosaccharides
PKC	Protein kinase C
PLC	Phospholipase C
RBD	Receptor binding domain
SCFA	Short-chain fatty acids
TcdA/B	TcdA/B
TEER	Transepithelial electrical resistance
TJ	Tight junction
