# Supplementary material for: Protective Effects of Alginate and Chitosan Oligosaccharides against Clostridioides difficile Bacteria and Toxin
Source: Toxins (Basel). 2023 Sep 22;15(10):586. doi: 10.3390/toxins15100586 (PMC10610568; doi:10.3390/toxins15100586)
Supplement: Supplementary file 1 [file toxins-15-00586-s001.zip › toxins-2155628-supplementary.pdf]

Supplementary Material

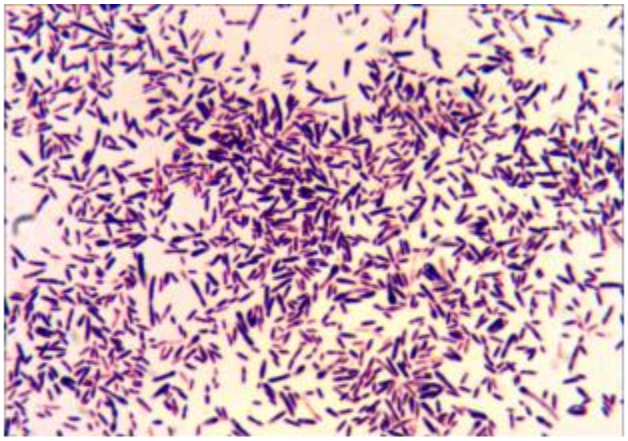

Figure S1. Gram staining of *Clostridium difficile*.

Rank (Quality)	Matched Pattern	Score Value	NCBI Identifier
1 (+++)	Clostridium difficile MB_4499_05 THL	2.13	1496
2 (+++)	Clostridium difficile MB_7476_05 THL	2.07	1496
3 (+)	Clostridium difficile 0422_0288_DM IBS	1.97	1496
4 (+)	Clostridium difficile MB_1562_05 THL	1.94	1496
5 (+)	Clostridium difficile MB_7869_05 THL	1.88	1496
6 (+)	Clostridium difficile MB_294_05 THL	1.85	1496
Result table for sample 89—continued on next page			
Result table for sample 89—continued from previous page			
7 (+)	Clostridium difficile MB_2559_05 THL	1.83	1496
8 (+)	Clostridium difficile DSM 1296T DSM	1.77	1496
9 (+)	Clostridium difficile 1020_NCTC 11206 BOG	1.77	1496
10 (+)	Clostridium difficile DSM 12057 DSM	1.75	1496

Figure S2. Computer display of identification results after automatic comparison of the generated spectrum with the MALDI-TOF database. The ten best matching entries are shown in a tabular form. The degree of similarity to the reference spectrum is represented by a score value. Identification results with score values above 2.0 are considered to be correct for determination of the respective species.

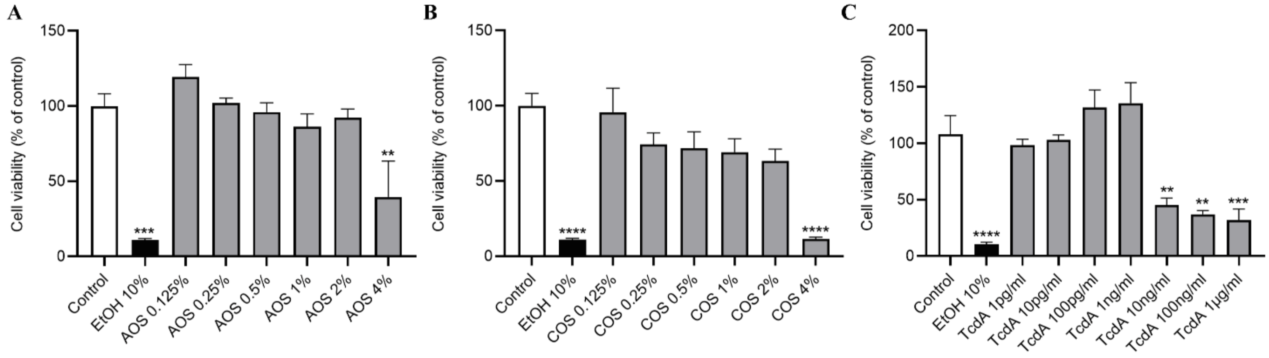

Figure S3. MTT cell viability assay. Caco-2 monolayers were grown in 96-well plates and exposed to (A) AOS and (B) COS 0.125%-4% for 48 h, or to (C) TcdA 1 pg/ml-1 µg/ml for 24 hours. DMEM and ethanol 10% served as positive and negative control, respectively. Results are expressed as percentage of positive control as mean ± SEM of three independent experiments, each performed in triplicate (**P ≤ 0.01, ***P ≤ 0.001, ****P ≤ 0.0001: significantly different from the unstimulated cells, as obtained using one-way ANOVA test).

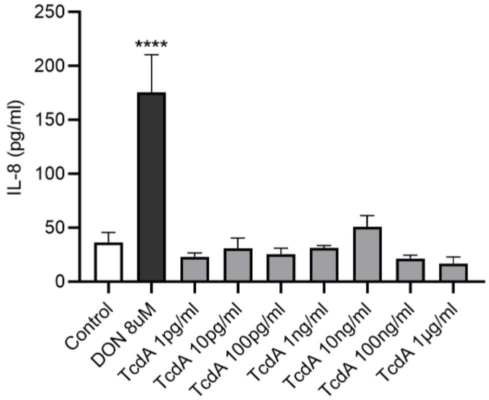

Figure S4. ELISA assay for IL-8. Incubation of Caco-2 monolayers with ascending TcdA concentrations did not induce IL-8 release. The toxin deoxynivalenol (DON) at a concentration of 8 µM and DMEM served as positive and negative control, respectively. Results are expressed as pg/ml of IL-8 released, as mean ± SEM of three independent experiments, each performed in triplicate (****P ≤ 0 .0001: significantly different from the unstimulated cells, as obtained using one-way ANOVA test).

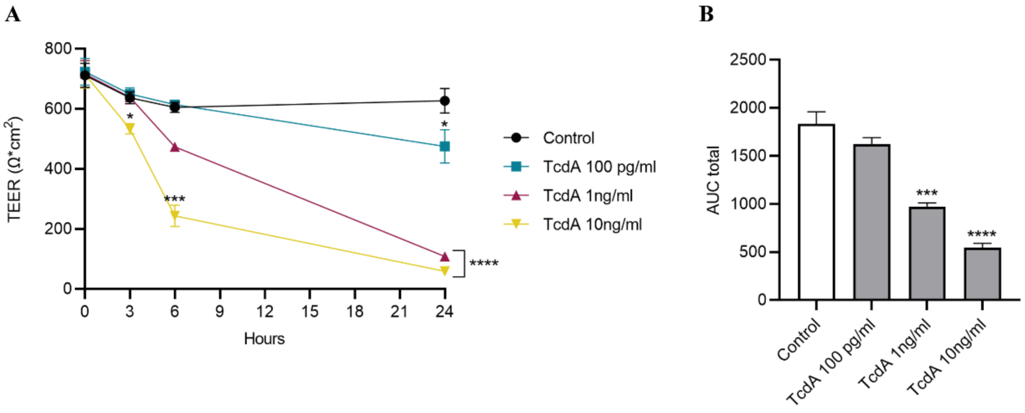

Figure S5. TEER trend and AUC of Caco-2 cells exposed to TcdA. Caco-2 cells grown in Transwell® inserts were exposed to TcdA 100 pg/ml, 1 ng/ml, and 10 ng/ml for 24 h. TEER values were measured prior to and after 6, 9, and 24 hours of TcdA exposure. (A) TEER trend (Ω*cm²) and (B) the total AUC for the time period of 24 h are presented as mean ± SEM of three independent experiments, each performed in triplicate (*P ≤ 0.05, ***P ≤ 0.001, ****P ≤ 0 .0001: significantly different from the unstimulated cells, as obtained using one-way ANOVA test, with TEER values (Ω*cm²) expressed as a percentage of the initial value).

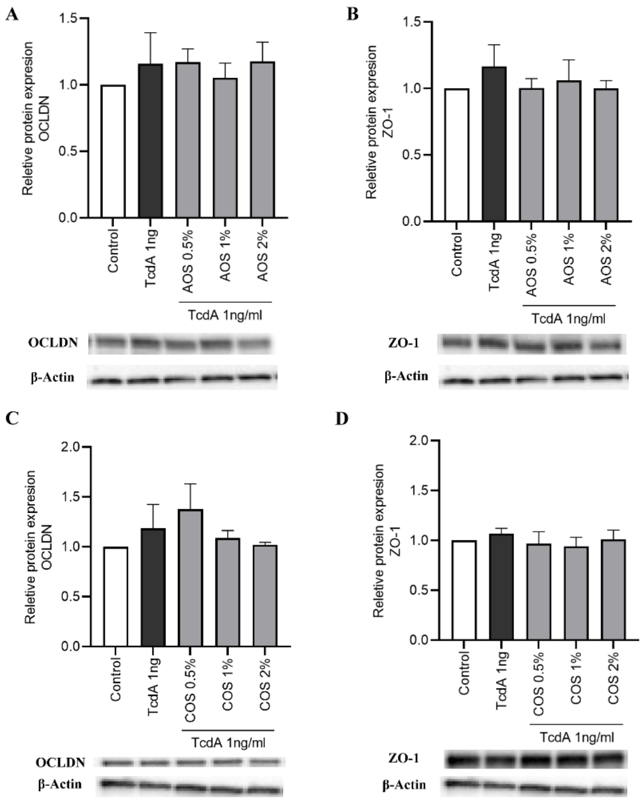

Figure S6. Relative TJ protein expression in response to TcdA and TcdA/NDOs. The effect of TcdA 1 ng/ml on the protein levels of ZO-1 and occludin were quantified by Western blot analysis in Caco-2 cell lysates of cells that had been exposed to the toxin for 24 hours, with or without total supplementation (pre- and post-TcdA challenge treatment) with increasing (A, B) AOS or (C, D) COS concentrations. Results are expressed as mean ± SEM relative protein expression of three independent experiments, each performed in triplicate. No significant (*P ≤ 0.05) difference was observed compared to unstimulated cells, as obtained using one-way ANOVA test.

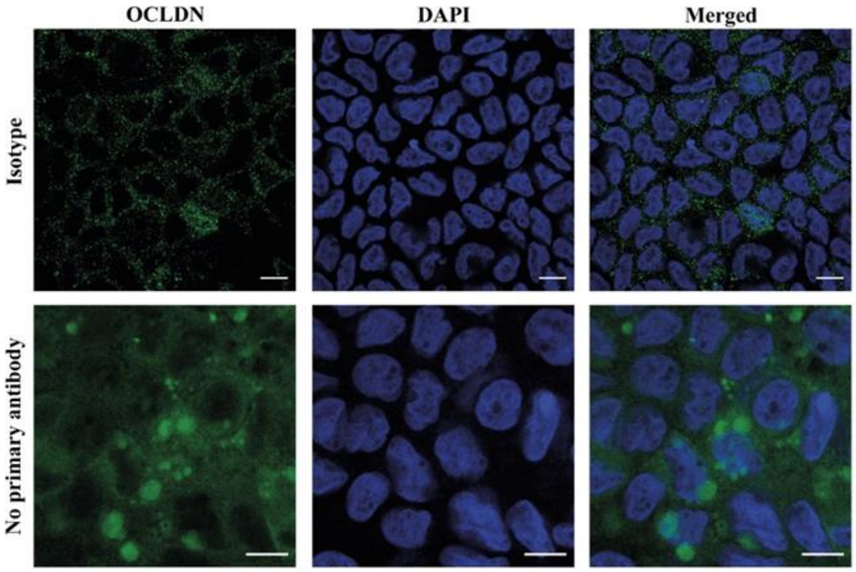

Figure S7. Isotype and negative controls for immunofluorescence staining. To differentiate non-specific binding from specific antibody signal, two staining controls were used (from top to bottom); an isotype control and a negative staining control, i.e., incubation with a secondary antibody only.

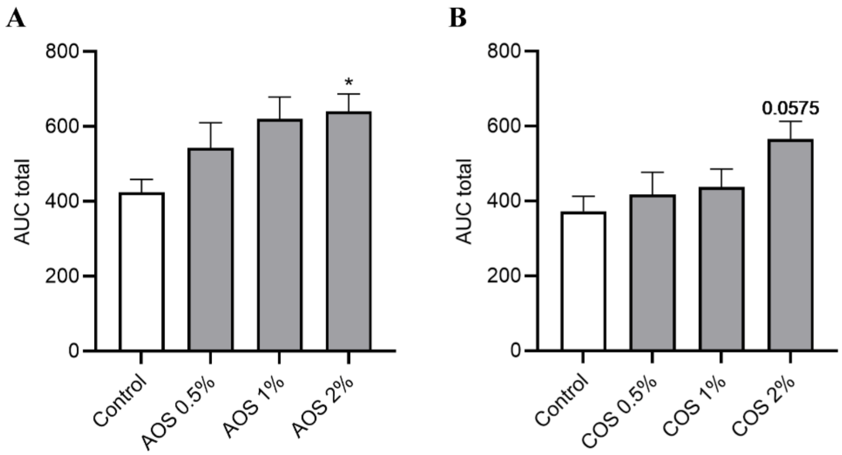

Figure S8. Total AUC for calcium switch assay upon AOS/COS treatment. To examine whether the NDOs accelerate TJ re-assembly, Caco-2 cells grown on Transwell® inserts were pre-treated with increasing AOS/COS concentrations (24 h) prior to transient calcium deprivation with HBSS-EGTA to disrupt intercellular contacts. TEER values were measured during recovery (0, 2, 4, 6, 8, 10 h) in complete, calcium-containing DMEM supplemented with either AOS (A) or COS (B). Results are expressed as the total area under the curve (AUC) calculated from TEER values for the time frame of 10h and expressed as the percentage of initial value as mean ± SEM of three independent experiments, each performed in triplicate (*$P \leq 0.05$: significantly different from the unstimulated cells, as obtained using one-way ANOVA test).
